# Assessing Disaster Preparedness among Latino Migrant and Seasonal Farmworkers in Eastern North Carolina

**DOI:** 10.3390/ijerph9093115

**Published:** 2012-08-30

**Authors:** Sloane Burke, Jeffrey W. Bethel, Amber Foreman Britt

**Affiliations:** 1 Department of Health Sciences, California State University, Northridge, CA 91330, USA; 2 College of Public Health and Human Sciences, Oregon State University, Corvallis, OR 97331, USA; Email: Jeff.Bethel@oregonstate.edu; 3 BSC USAFA, United States Air Force, Colorado Springs, CO 80911, USA; Email: uncamfo@gmail.com

**Keywords:** disaster preparedness, Latino migrant and seasonal farmworkers, MSFW

## Abstract

Natural disasters including hurricanes, floods, earthquakes, tornadoes, and fires often involve substantial physical and mental impacts on affected populations and thus are public health priorities. Limited research shows that vulnerable populations such as the low-income, socially isolated migrant and seasonal farmworkers (MSFW) are particularly susceptible to the effects of natural disasters. This research project assessed the awareness, perceived risk, and practices regarding disaster preparedness and response resources and identified barriers to utilization of community and government services during or after a natural disaster among Latino MSFWs’ and their families. Qualitative (N = 21) focus groups (3) and quantitative (N = 57) survey methodology was implemented with Latino MSFWs temporarily residing in rural eastern North Carolina to assess perceived and actual risk for natural disasters. Hurricanes were a top concern among the sample population, many participants shared they lacked proper resources for an emergency (no emergency kit in the house, no evacuation plan, no home internet, a lack of knowledge of what should be included in an emergency kit, *etc*.). Transportation and language were found to be additional barriers. Emergency broadcasts in Spanish and text message alerts were identified by the population to be helpful for disaster alerts. FEMA, American Red Cross, local schools and the migrant clinic were trusted places for assistance and information. In summary, tailored materials, emergency alerts, text messages, and news coverage concerning disaster threats should be provided in the population’s native language and when feasible delivered in a culturally appropriate mechanism such as “charlas” (talks) and brochures.

## 1. Introduction

An estimated 3–12 million migrant and seasonal farmworkers (MSFWs) currently reside in the United States (US), the majority of which are Latinos. North Carolina (NC) is a top agricultural state and ranks sixth in the nation for migrant farmworker population, with approximately 200,000 MSFWs. MSFWs are a transient, invisible population and therefore, accurate estimates of the population are hard to gauge. The vast majority of this population lives below the poverty level, with half earning less than $7,500 annually [1]. Overall, MSFWs comprise nearly 50% of all hired farm workers in the US, are considered minority groups in society and are usually foreign born—the majority coming from Mexico or Central America [1,2,3,4,5]. MSFW are uninsured or underinsured employees in a multi-billion dollar agricultural industry [6]. 

The low-income MSFW population, along with other vulnerable groups, are particularly susceptible to the effects of natural disasters [2,3,4,6,7,8,9,10,11,12,13,14]. Their location in rural areas that already lack needed resources is further confounded by literacy issues leading to heightened risk—with marginalized groups being disproportionally affected by extreme weather events [15,16,17]. The National Atlas shows that NC is a state with high rates of hurricane landfalls and extreme heat events [18]. On 16 September 1999, Hurricane Floyd, a category-two storm with wind speeds of 110 mph, made landfall at Cape Fear, NC. Its rains accompanied by high inland water levels from Hurricane Dennis (which struck the preceding week) led to unprecedented flooding that in the words of past governor Jim Hunt “was the worst disaster to hit NC in modern times” causing 52 deaths, damaging 24,000 homes, and displacing 47,000 residents [19,20,21]. Natural disasters are a clear and present threat in NC and, while MSFWs have been identified as a population particularly susceptible to the effects of a disaster, there has been little research aimed at this group. The majority of previous qualitative or quantitative assessments examining the relationships between social and economic factors and weather-related risks have been conducted among vulnerable populations as classified by race/ethnicity, immigration status, or social-economic status [12,19,20,22,23,24,25,26,27,28,29,30,31,32,33,34,35,36,37]. While MSFWs fit into these broader classifications, few studies have addressed the needs and challenges specific to this group. 

One of the quintessential issues for public health preparedness planning is how to motivate people to prepare for disasters. This matter becomes further complicated when the target population is Latino immigrants, particularly because there is a paucity of information available regarding the knowledge, attitudes, and beliefs about disaster preparedness among low-income immigrants in the US. Shiu-Thorton *et al*. made the grave prediction that “without clear and proactive planning to strategically meet the needs of limited English proficient (LEP) communities, disaster scenarios will have adverse effects for LEP groups, deepening the health disparities that already exist for those populations”. [23]. Similarly through qualitative interviews, medical interpreters found that preparedness was not a concept for many non-native English speakers—they simply did not discuss the potential for disasters or engage in community discussion concerning disaster preparedness [24]. 

Studies have shown that prior experience with emergencies influence one’s response to a new emergency situation. Bolin found that Mexican immigrants who experienced the Mexico City earthquake responded very differently to the Northridge, California earthquake than those without the prior disaster experience [25]. Some immigrants who had survived wars and civil conflict in their native lands “believe that America is a safe place; therefore, there is no need to prepare” [23]. However, Eisenman *et al*. found that Latino participants with prior experience with disasters prompted them to make some preparations for future disasters [22]. These apparent discrepancies underscore the need for further research into the perceptions and beliefs of different cultural communities in order to develop culturally competent and materials and effective disaster management plans. 

Minorities are less likely to feel prepared for an emergency and to have an emergency plan than the general public [26]. MSFWs are at a unique disadvantage due to the lack of understanding about their risk perception as well as language and literacy barriers; therefore, public health efforts should strive to tailor messages that address the distinctive needs and characteristics of this vulnerable population [24]. Previous research using Latino focus group interviews found that small group discussions (*platicas* or *charlas*) are the participants’ preferred method to learn about disaster preparedness [22,26,37]. A cohort study revealed larger improvements in preparedness from participants who were assigned to attend *platicas* than those who received a culturally tailored mailer [37]. 

While it is obvious that more studies on this public health issue among disadvantaged groups are needed, conducting research in the MSFW population is challenging. For MSFWs, anti-immigrant legislation coupled with the undocumented status of many MSFWs has resulted in a lack of trust in assessments as well as in the organizations performing the assessments. The end result has been a decrease in their participation in research efforts. Conducting a population-based survey is a vital component to the continued assessment of this issue. A review of the literature yielded no quantitative data instrument specific to MSFWs and their assessment of disaster preparedness. 

Eisenman *et al*. concluded that community engagement, culturally competent approaches, participatory methods, and partnerships among universities, public health agencies, and community-based organization are broadly recommended over macro-level risk-communication practices [37]. The community-based participatory research (CBPR) methodology employed in this study was developed in collaboration with MSFW communities, who in turn have empowerment and ownership of the research and recommendations, thereby increasing the likelihood of success of disaster preparedness plans for this population [38]. This approach, capitalizing on shared cultural valuing of children, family, and community and their social networks rather than focusing on the individual response to reaching Latino communities with disaster preparedness interventions has been shown to be important by the limited number of extant studies among Latinos [23,24,27]. 

In recognition of the particular vulnerabilities of MSFWs and the risk of natural disasters in NC, particularly in low-lying coastal areas, the objectives of this study were to determine: (1) the level of awareness and perceptions of risk regarding natural disasters among Latino MSFWs; and (2) the awareness, use, and barriers, if any, to available community resources during and after natural disasters among Latino MSFWs. 

## 2. Experimental Section

### 2.1. Methods: Stakeholder Meeting and Interviews

An essential part of CBPR methodology is the engagement of community partnerships in the preliminary stages of the research design. Researchers convened a meeting with key stakeholders as the first phase of the project to establish trust and elicit interest in the project. The stakeholders included key informants, community members, agency and healthcare providers, members of grassroots organizations and academic partners from a neighboring university. These established partnerships were instrumental in building project collaboration and developing a research plan that would work best for the community while establishing trust with key community members. 

Participants were guided through a series of questions about currently available resources, the perceived awareness regarding preparedness among MSFWs, channels of communication, barriers experienced by the MSFWs, and effective methods of relaying information. They were also probed for ideas that service providers could use to work together to provide support to MSFWs prior to and in the event of a natural disaster. A member of the research team transcribed the audio taped meeting and a thematic analysis of the content was performed. Participants unable to attend the stakeholder meeting were contacted by a member of the research team for a follow-up interview. A virtual stakeholder meeting was held during project wrap-up to encourage continued collaboration and report findings from the focus group and quantitative survey instrument.

### 2.2. Focus Groups

During the spring and early summer of 2010, participants were recruited from an county in eastern NC which has some of the highest estimated populations of MSFWs in the state and nation [1,7,39]. Any male or female MSFW or someone living with a MSFW aged 18 or older and residing in the specified county were eligible to participate in the study. Adult family members of the MSFWs were included in the study because research in the general population has found that wives or caregivers are usually the ones that value and implement disaster preparedness programs for their children and families [33,39]. Furthermore, research shows that Latino males are more likely to prepare for the benefit of protecting their families than for self-preservation [27]. A trusted community gatekeeper recruited participants via “word-of-mouth” at migrant camps and a local clinic that serves this community. 

A total of three focus group interviews were conducted at a community health clinic, a convenient and trusted location for those who were participating. These sessions were conducted in Spanish by two trained bilingual facilitators. The instrumentation for the qualitative focus groups represented a compilation of ideas arising from the stakeholder meeting and a thorough review of the literature. At the beginning of each focus group session, verbal informed consent was obtained from all participants. The University Medical Center’s Institutional Review Board approved the use of verbal consent in recognition of the vulnerability of this population and possible literacy issues and to ensure the anonymity of potentially undocumented residents. 

The bilingual facilitator followed a semi-structured format, using a series of 20 open-ended questions developed by the research team to guide and encourage discussion of the term “natural disaster”, the concept of preparedness, actual supplies on hand, resources and barriers, as well as trusted sources of information and aid. During the course of the focus group interviews, the facilitator probed the participants’ responses whenever necessary to further explore the topics and elicit deeper discussion. 

The audio taped discussions from each focus group were transcribed verbatim by a bilingual member of the research team. These transcripts were then translated into English and back-translated to ensure accuracy and validity. The transcripts were synthesized with the notes taken during the interviews to capture body language cues and small nuances not conveyed in the audio taped discussion. The final form of the transcripts was analyzed by two members of the research team trained in qualitative research methodology. Transcripts were read and independently coded by assigning coding themes to appropriate segments of text. Inter-rater reliability was established by the pair through an iterative process where coders went through the interview transcript and coded it. Codes for specific interviewers were compared to check for inter-rater reliability when the pair met to discuss discrepancies in coding and reach consensus. This process was repeated until at least 90% consensus was achieved within the pair of independent coders. 

### 2.3. Quantitative Survey Development and Testing

The instrument developed for this study relied on the results from the three focus groups, the stakeholder meeting, and the knowledge gleaned from the literature review. Additionally, published disaster preparedness surveys aimed at the general public were adapted as necessary by the research team to be appropriate and culturally competent for the MSFW community. 

The survey was developed in English and then translated by a fluent member of the research team. The content and construct validity were reviewed by a native Spanish speaker, a disaster preparedness expert, and an expert on Hispanic culture. The questionnaire was written at a maximum of fifth grade level and participants had the option of an oral administration of the survey due to the literacy issues within the population. It included thirty-four close-ended items describing demographics, self-identified language ability and communication, previous experience with disasters, general preparedness including actual supplies on-hand, motivation for preparing, barriers to preparedness, trusted sources of information, and preferred methods of receiving information. Descriptive statistics were used to examine the individual items collected in the survey using Version 18.0 of PASW for Windows [40]. 

A mixture of migrant and H2A workers (n = 46) and seasonal farmworkers (n = 11) were recruited to participate in the study. A $10 gift card was provided as incentive for participating. Three bilingual community gatekeepers as well as a bilingual member of the research team assisted in administering the instrument by circulating around the room and asking participants if they needed one-on-one assistance. One trusted community gatekeeper read the survey items aloud to the entire group. Prior to the administration of the instrument, the informed consent statement was read by same gatekeeper in Spanish and all members agreed to participate. The farmworkers were asked not to put any identifiable information on the surveys and all responses were kept anonymous. 

## 3. Results

### 3.1. Stakeholder Meeting and Interviews

Joining the four member research team at the initial stakeholder meeting were six stakeholders including a representative from the North Carolina Office of Rural Health & Community Care who served as the main consultant to the Migrant Health Program, the Disaster Preparedness Coordinators from two local health departments, the Director of Migrant Outreach for the county’s migrant health clinic and two faculty members from a neighboring university with expertise in geography and disaster preparedness, respectively. Follow-up interviews were performed including a face-to-face interview with a representative from the Red Cross. 

One of the stakeholders shared his experiences working with migrants during the flooding caused by Hurricane Floyd. Several items discussed during the meeting included the possibility of using a reverse-911 system with a Spanish message or cell phone text messages to alert migrant workers of severe weather, and the logistics and feasibility of getting migrant farmworkers added the county Emergency Disaster plans. Several stakeholders spoke of the difficulty of disseminating information to the monolingual Latino farmworkers, namely the inability of the National Oceanic and Atmospheric Association to broadcast an alert in Spanish, the lack of real time Spanish language channels, and fact that local news programs are only in English. The county emergency managers stressed their need for accurate information about the density and location of MSFWs, especially those who are located in floodplains; however there were concerns expressed about having this information be used by Immigration and Customs Enforcement. 

One of the Disaster Preparedness Coordinators acknowledged that there was a previous ten question disaster preparedness survey developed by a neighboring health department that had been translated into Spanish but it had never been evaluated for cultural competency. Now that key stakeholders had been identified, the consensus of the group was to encourage future collaborations to ensure that MSFW are integrated into the county’s disaster response plan and that an assessment tool evaluating the farmworkers’ knowledge, attitudes, and practices regarding natural disasters was of paramount importance. 

### 3.2. Focus Group Interviews

*Participant characteristics. *Two groups were composed of seasonal workers (n = 14) while the other group was a mix of migrant and H2A workers (n = 7). All of the participants in the migrant worker group were male (100%) and approximately 86% of them were married and had children under the age of 18 living in their household (Table 1). The average age of the migrant participants was approximately 37 years old with a range from 28 to 42. Approximately 57% of the seasonal workers were female and roughly 71% of them were married. Children under the age of 18 were present in approximately 64.3% of the seasonal workers’ households. The age of the seasonal workers ranged from 22 to 43 years old, with an average of approximately 31 years. The seasonal workers have lived in the United States for an average of approximately 10 years. 

**Table 1:** Demographic characteristics of focus groups participants.

Characteristic		Migrant workers	Seasonal workers
		(7 participants)	(14 participants)
		%	%
Sex			
	Male	100.0	42.9
	Female	0.0	57.1
Age (years)			
	20–29	14.3	46.2
	30–39	57.1	38.5
	40–49	28.6	15.4
Marital status			
	Married	85.7	71.4
	Not Married	14.3	28.6
Child (≤18) present in household			
	Yes	85.7	64.3
	No	14.3	35.7
Total seasons/years in the United States			
	≤5	----	14.3
	6–10	----	42.9
	11–15	----	42.9

*Emergent themes*. The main findings developed from the analysis of the focus groups are provided in six sections: (1) Hurricanes are a top concern; (2) Resource availability and preparedness item; (3) Motivation for preparedness; (4) Barriers to resources; (5) Preferred sources and types of information; (6) Effects of a natural disaster. The interview guide can be found in Appendix 1.

*Hurricanes are a top concern*. Participants spoke about what the term “natural disaster” meant to them. In each of the focus groups, the term “hurricane” came up almost immediately. Additionally, tornadoes, tsunamis, floods, earthquakes and snowstorms were often cited. Many participants, both migrant and seasonal, had previous experience with hurricanes in NC. For example: “Floyd, I will never forget that name. In 1996 there was one and then Floyd. But Floyd was the worse; it flooded everything where we lived”. Few of them had experiences with natural disasters in Mexico, but at least one participant provided an explanation for this: “During the times when natural disasters occur is the time that we are here more than we are there (Mexico). We are there during the time when there aren’t many disasters”.

*Resource availability and preparedness items*. Several of the MSFWs had a general idea of the types of items they needed to prepare for a natural disaster. Participants mentioned: candles, flashlights, canned food, potable water, a radio with a battery, first-aid kit, and blankets, but few, if any, had these items in their house. Overall, the participants reported being grossly underprepared for a natural disaster and that they did not have access to the internet, emergency kits, evacuation plans or a written explanation of procedures on what to do if a disaster occurred. One respondent reported having an emergency kit but admitted that is was seriously inadequate and only had enough supplies for one person. Interestingly, another participant explained that she had not ever been told about how to prepare for a disaster; the only thing that she knew was that she was supposed to bring her “important papers” (documents that prove she is here legally) but nothing else. A participant in a different focus group indicated that one should have “passports and important papers. You should put them in bags, in plastics things” to protect them. Their children and one trusted community member (a worker at the local community health center) were cited as great resources especially because of their bilingual language ability.

*Motivation for preparedness.* When asked if they thought that preparedness was important, all of the MSFWs replied affirmatively. One of the biggest components of this theme was that it was important to be prepared for your family or for your children. One of the migrant workers, summed it as: “Okay the motivation I believe is the obligation that each one brings from the moment he leaves Mexico that they came to work and he comes motivated to achieve something and to do for your family, the risk is always going to be but the motivation has to come through the family, that one tries to run the least risk possible”. 

*Barriers to resources*. The MSFWs identified barriers to accessing resources both before and during the event. Consistently, one of the more common barriers was language—there are no local public Spanish radio or television programs and alerts are broadcasted only in English. They agreed that materials, alerts, and general information need to be provided in Spanish. Financial constraints were also noted, especially concerning discussions about preparedness materials and emergency kits. Farmworkers in two of the three focus groups listed transportation as an issue. Additionally, one of the participants indicated that lack of knowledge was a significant barrier: “(The alert is) an alarm when a disaster is coming. The only problem is that then they announce where it’s coming from but because we don’t know the area we go where the disaster is. We don’t know our surroundings, (we need). to know the names of the states to know where the disaster is coming from; there isn’t a way to go and find it. Many times we have the opportunity that the growers give us to leave, but because we don’t know where the problem is coming from we leave and run into it and we really need to know this”. 

*Preferred sources and types of information*. In general the farmworkers had a positive attitude about local governmental and nongovernmental sources of information and aid and felt they would feel confident seeking aide/assistance in the event of a natural disaster. The migrant outreach program director was cited in all three focus groups as being a trusted source of information along with the police, American Red Cross, firefighters, the military (National Guard), growers, radio, and television new shows. Participants stated that schools were a great source because they sent home information about the disaster with their children and there were several trusted bilingual individuals that worked at the school. When asked about the type of information that they preferred, first and foremost they stated the information needs to be provided in Spanish. One participant stated that emergency information and alerts should be translated, “what they say in English, they should say in Spanish as well”. They thought that drills, personal testimonies from a Latino who had been through the experience, brochures, and short television specials/videos would be the most effective way of communicating disaster preparedness information. 

*Effects of a natural disaster*. The greatest problem in the aftermath of a natural disaster were fear and loss of a relative. Although many people stated that losing their houses and belongings would be difficult, especially since they did not have insurance, several people thought that death was more important. As one participant stated, the greatest problem in the aftermath of a weather-related disaster, would be to “lose a relative, because things come and go. We would begin again from the bottom”. Additionally, the MSFWs thought that loss of power, a disruption of communication systems, and transportation problems were other potential detrimental effects. Another participant noted that it would be very difficult for them if all the crops were destroyed, “…if it’s time to harvest the crops and it’s a water-related natural disaster everything would be lost. Then immediately our contract would end because there would be no harvest for us”. 

These six emergent themes were synthesized and then incorporated along with existing disaster preparedness questionnaires and a subset of questions from the Behavioral Risk Factor Surveillance Survey (BRFSS) into a culturally competent quantitative survey instrument [41]. To our knowledge no disaster preparedness instrument exists specific to the literacy levels and unique needs of the MSFW population. Therefore, the feedback from the focus group interviews was instrumental in adapting specific items from existing preparedness surveys, particularly potential answers to closed ended survey items, into a form that was applicable to this group.

### 3.3. Survey

A mixture of migrant and H2A workers (n = 46) from a migrant farmworker camp and seasonal farmworkers (n = 11) completed the pilot survey. Among migrant and H2A workers, analyses revealed that all respondents were male and from Mexico (Table 2). The mean age of the farmworkers was 36 years and with a range from 20 to 60 years. Approximately 89% of the participants were married or living as married. Approximately 96% of the workers self-identified their ability to speak English as “not at all” or “a little” and no one reported speaking English well. The migrant and H2A group had lived in the US for an average of roughly 11 years. When asked about previous experience with natural disasters, approximately 53% responded that they were residing in NC when Hurricane Floyd struck in 1999.

Among seasonal farmworkers, the majority of the sample was female (72.7%) and approximately 91% were from Mexico (one was born in the US) (Table 2). The age range of the seasonal farmworkers was 22–68 years with a mean of approximately 41 years. Approximately 64% of the seasonal workers were married or living as married and 27% had children under the age of 18 present in their U.S. household. The self-reported language ability was higher than the mixed migrant and H2A worker group with approximately 64% of the farmworkers self-reporting speaking English “somewhat” or “well”. Roughly 55% of the workers were residing in NC when Hurricane Floyd struck in 1999. This sample of seasonal farmworkers had lived in the United States for an average of 13.4 years.

**Table 2:** Demographic characteristics of survey respondents.

Characteristic		Migrant workers	Seasonal workers
		(n = 46)	(n = 11)
		%	%
Sex			
	Male	100.0	27.3
	Female	0.0	72.7
Age (years)			
	20–29	26.1	36.4
	30–39	41.3	9.1
	40–49	19.6	36.4
	≥50	13.0	18.2
Marital status			
	Married/Living as Married/Widowed	88.6	63.6
	Not Married	11.4	36.4
Child (≤18) present in household			
	Yes	73.3	27.3
	No	26.7	72.7
Self-identified ability to speak English			
	Not at all	50.0	0.0
	A little	45.7	36.4
	Somewhat	4.3	45.5
	Well	0.0	18.2
Total seasons/years in the United States			
	≤5	10.9	9.1
	6–10	28.3	18.2
	11–15	45.7	45.5
	≥16	15.2	27.3
Lived in NC during Hurricane Floyd (’99)			
	Yes	52.6	54.5
	No	47.4	45.5

In general, the migrant farmworkers expressed a higher degree of concern about specific natural disasters than did the seasonal workers. The seasonal workers polled only expressed being extremely concerned over two weather events whereas migrant workers expressed being were extremely concerned about all eight of specified weather events (Table 3 and Table 4). Hurricanes and floods were the exception for this trend where 100% of the seasonal workers were concerned, very concerned, or extremely concerned about being affected by a hurricane and only 75.6% of migrants shared the same degrees of concern over hurricanes. Nearly 73% of the seasonal workers were concerned, very concerned, or extremely concerned about floods compared to 66.6% of the migrant workers who shared this opinion.

**Table 3:** Migrant farmworkers’ degree of concern about specified natural disasters affecting their community. n = 46 Frequency (Valid %).

Natural Phenomenon	Not Concerned	Somewhat Concerned	Concerned	Very Concerned	Extremely Concerned
Hurricanes	2 (5.4%)	7 (18.9%)	9 (24.3%)	10 (27.0%)	9 (24.3%)
Extreme Heat	---	5 (15.2%)	7 (21.2%)	9 (27.3%)	12 (36.4%)
Drought	2 (6.5%)	7 (22.6%)	5 (16.1%)	7 (22.6%)	10 (32.3%)
Earthquake	7 (22.6%)	4 (12.9%)	4 (12.9%)	3 (9.7%)	13 (41.9%)
Flood	4 (13.3%)	6 (20.0%)	6 (20.0%)	1 (3.3%)	13 (43.3%)
Wild Fire	7 (23.3%)	6 (20.0%)	5 (16.7%)	4 (6.7%)	10 (33.3%)
Tornado	3 (9.7%)	2 (6.5%)	12 (38.7%)	4 (12.9%)	10 (32.3%)
Winter Storm (snow or ice)	11 (35.5%)	6 (19.4%)	10 (32.3%)	---	4 (12.9%)

**Table 4:** Seasonal farmworkers’ degree of concern about specified natural disasters affecting their community. n = 11 Frequency (Valid %).

Natural Phenomenon	Not Concerned	Somewhat Concerned	Concerned	Very Concerned	Extremely Concerned
Hurricanes	---	---	2 (18.2%)	5 (45.5%)	4 (36.4%)
Extreme Heat	2 (18.2%)	5 (45.5%)	2 (27.3%)	1 (9.1%)	---
Drought	4 (36.4%)	6 (54.5%)	1 (9.1%)	---	---
Earthquake	3 (27.3%)	7 (63.6%)	1 (9.1%)	---	---
Flood	---	3 (27.3%)	2 (18.2%)	2 (18.2%)	4 (36.4%)
Wild Fire	1 (9.1%)	10 (90.9%)	---	---	---
Tornado	1 (9.1%)	4 (36.4%)	5 (45.5%)	1 (9.1%)	---
Winter Storm (snow or ice)	4 (36.4%)	4 (36.4%)	3 (27.3%)	---	---

A majority of the migrant and H2A workers reported having some disaster preparedness items on hand—73.3% had a three day supply of water, 71.1% had blankets, 60.0% had a flashlight, and 57.8% had a battery-operated radio. The preparedness item least likely to be in their possession was a 3-day supply of medicine (26.7%). Only 11% felt that their household was well prepared for a natural disaster. Approximately 84% of the all respondents reported keeping their family safe as their motivation for preparing for a disaster; roughly 67% cited keeping themselves safe and 60% were motivated to protect their home and their belongings (Table 5). The Red Cross (67%), police (65%), and firefighters (65%) were the most often cited sources from which the workers could receive emergency information in the event of a natural disaster. They preferred to receive emergency alerts from the radio (91%), television (78%), and via cell phones or text messages (58%). The majority of the workers reported having a cell phone with service at their home (81.8%) and at their work site (74.4%). The barracks style camp where all the workers lived did not have a computer or access to the internet. 

**Table 5:** Frequencies of selected characteristics from the quantitative survey instrument.

Characteristic		Migrant workers	Seasonal workers
		(n = 46)	(n = 11)
		Frequency (%)	Frequency (%)
Motivation to prepare			
	Keeping my family safe	38 (84.4%)	11 (100%)
	Keeping myself safe	30 (66.7%)	9 (81.8%)
	Protecting my home and belongings	27 (60.0%)	10 (90.9%)
Trust source of information			
	Red Cross	31 (67.4%)	1 (9.1%)
	Police	30 (65.2%)	2 (18.2%)
	Firefighter	30 (65.2%)	---
	Clinic	17 (37.0%)	6 (54.5%)
	Grower	16 (34.8%)	3 (27.3%)
	Church	8 (17.4%)	3 (27.3%)
	Outreach worker	8 (17.4%)	10 (90.9%)
	Public Health Department	7 (15.2%)	---
	School	5 (10.9%)	7 (63.6%)
	Latino festivals	4 (8.7%)	---
	FEMA	1 (2.2%)	---
Barriers			
	Lack of information in Spanish	37 (84.1%)	11 (100%)
	Lack of knowledge where natural disaster is located	28 (63.6%)	11 (100%)
	Lack of knowledge of what to do	27 (61.4%)	10 (90.9%)
	Lack of transportation	17 (38.6%)	10 (90.9%)
	Lack of knowledge of current location	13 (29.5%)	9 (81.8%)
Greatest concern following a natural disaster			
	Death of a family member	40 (93.0%)	11 (100%)
	Losing house	30 (69.8%)	11 (100%)
	Losing job	29 (67.4%)	6 (54.5%)
	Losing touch with family	28 (65.1%)	9 (81.8%)
	Losing belongings	24 (55.8%)	9 (81.8%)
	Being overwhelmed	8 (18.6%)	2 (27.3%)

The most significant barrier in accessing emergency services or information cited by the migrant workers was a lack of information in Spanish (84%), followed by not knowing the actual location of the disaster (64%), and not knowing what they should do (61%). Only 37% of the group had received information on how to prepare for a natural disaster. When asked about their preferred method of receiving disaster preparedness information, the top three methods were: television (61%), radio (58%), and videos (44%). Losing a family member (93%), losing their house (70%), losing their job (67%) and losing their belongings (56%) were their principle concerns in the event of a natural disaster.

Overall, the seasonal workers were better prepared for a disaster with 82% having blankets, 73% having a battery operated radio, and 64% having a flashlight as part of their emergency preparedness kit. None of those surveyed reported having a 3-day supply per person of food and only 18% reported having a 3 day supply of water per person. Approximately 11% felt that their household was well prepared for a natural disaster. All of the seasonal workers felt that their motivation to prepare for a disaster came from keeping their family safe, 91% were motivated by keeping their homes and belonging safe, and 82% found motivation in self-preservation (Table 5). The top three preferred sources of emergency information were outreach workers (91%), schools (64%) and the local health clinic (55%). The radio was cited by each member of the group as the preferred method of receiving emergency alerts, with television (64%), family/friends (46%) and cell phones/text messages (46%) being other preferred methods. 91% of the workers had a cell phone with service at their homes and 81.8% also reported having service at their work sites.

Lack of information in Spanish (100%) and knowing the location of a natural disaster (100%) were the most significant barrier to accessing emergency services and information during a natural disaster. Other barriers included not knowing what to do (91%) and concerns about transportation (91%). Only 18% reported having received information on preparedness but 91% stated that they would like to receive this information via brochures, 82% indicated that they would prefer videos and 73% cited a preference for the radio. In the event of a natural disaster, their principle concerns were losing a family member (100%), losing their house (100%), losing their belonging (82%) and losing touch with their family (82%). The complete survey can be found in Appendix 2.

## 4. Discussion

The purpose of this study was to assess the knowledge, attitudes and perceptions concerning disaster preparedness among Latino MSFWs which then could be utilized to create an appropriate disaster preparedness response plan for this community. Consistent with the CBPR methodology, each step of the process included the participation and feedback from community stakeholders and the MSFW population. Each phase of the process built the foundation for the subsequent phase and therefore, the quantitative survey instrument that was one of the main tangibles of the project was both valid and robust.

The participants in both the qualitative and quantitative phases of the project felt that preparedness was important although they admitted that they did not know how to prepare or lacked needed preparedness items. This is in contrast to the results from a previous study which found that preparedness was not a concept for many non-native English speakers [24]. Participants who had experienced Hurricane Floyd were more likely to make preparations or be more aware of preparedness than those who had never experienced a natural disaster, reaffirming conclusions from the Bolin and Eisenman studies [22,25]. 

### 4.1. Stakeholder Meeting and Interviews

The participation of several key community stakeholders underscores the commitment to and concern for the MSFWs. Many of the response organizations were eager to work with this population but lacked either the language skills or the cultural competence to know how to most effectively reach them. Throughout the project, members of the stakeholder group collaborated to produce several tangible deliverables including Geographic Information System (GIS) maps of the publically registered H2A camps and their location within the flood plain and feedback on the quantitative survey instrumentation before pilot testing with the farmworkers. Holding true to the principles of CBPR, one of the biggest accomplishments of this phase of the research project was the commencement of dialogue between key community stakeholders and the realization of those at the county level of the importance of including the farmworkers in disaster response plans as well as a recognition of the special needs, vulnerabilities, and barriers faced by this population.

### 4.2. Focus Group Interviews

Several interesting and unique findings emerged from the analysis of the themes from the focus groups. Many of the participants had prior experience with hurricanes in NC. They were able to offer suggestions as to the best way to reach other Latinos and encourage them to become prepared for the possibility of a natural disaster. Previous studies show ambiguous results concerning the effect of prior experience with disasters and the level of preparedness for future disasters and further research is warranted [22,23,25]. Even though several of the workers seemed to have a general understanding of what supplies were needed in the event of a natural disaster, more work is needed to translate this knowledge into actual preparedness activities. Barriers identified by the participants should be studied and measures must be undertaken to ensure this vulnerable population is prepared for a future disaster event. The researchers were surprised to discover that government agencies such as the American Red Cross, FEMA, public schools, and the local migrant clinic were trusted resources among seasonal workers (not migrants) during a time of disaster. This information was provided in response to the interview question, “Are you aware that there are private and government aid organizations that respond to weather-related disasters? Can you name any of them?”. It appears the responses may be due to the positive experience with these agencies during Hurricane Floyd in 1999. 

Consistent with the study by Messias *et al*., MSFWs were motivated by their family to both accomplish everyday tasks and prepare for disasters [27]. Not surprisingly, the biggest barrier in accessing services and preparing for disasters was limited English proficiency. Disaster alerts are only broadcast in English in Eastern North Carolina. One participant discussed that they did not know their geographical position relative to reported disasters. She stated that when an alert is broadcast, they could evacuate towards the disaster instead of fleeing from it. In response to this deficiency, hurricane tracking maps will be provided for posting in migrant camps. Further understanding their preferred sources and types of information as well as knowing what they perceive as the worst effect of a natural disaster will enable emergency planners and outreach workers to tailor disaster plans to the MSFW community. Although these themes are informative, further engagement with the community needs to be conducted with a more diverse and representative sample to ensure saturation of themes and ideas. 

### 4.3. Survey

There was an apparent difference between the responses from the seasonal farmworkers and the migrant farmworkers—especially in terms of trusted sources of information and barriers. While differences in some items (e.g., self-reported language ability was higher among seasonal workers than the migrants) were not surprising, the results of this pilot study seem to denote that there are distinct differences in preferences, attitudes, and practices between the migrant and seasonal groups. Further research is needed to determine if these apparent differences remain in a larger and more representative sample. If these variances persist, separate assessment instruments and tailored plans should be developed that target the migrant and the seasonal groups’ specific preferences and challenges. 

In response to the question about perceived concern about disasters occurring in NC, several of the migrant workers listed being concerned to very concerned about an earthquake (Table 4). NC does not lie in an earthquake fault zone, but the study was conducted in the aftermath of the devastating earthquake in Haiti which could have caused the MSFWs to overestimate the likelihood of experiencing this natural phenomenon. The timing of the study along with the seasonal migration patterns of the workers (who are mainly in the United States from March to November) could also contribute to the low perception of severity concerning winter storms cited by the migrant farmworker group. However, this is an indication of an effective strategy that could be employed by preparedness workers centers along with using media coverage of current international disasters to educate and encourage their own citizens to be prepared for a disaster. 

### 4.4. Limitations and Strengths

One of the major limitations of this study was the potential for selection bias due to the small sample size of the pilot study. There was a larger than normal percentage of female, limited English-speaking participants for the focus group and for the pilot testing of the survey instrument with the seasonal workers. One explanation for this result is that this area of NC employs a substantial number of female seasonal workers who work primarily in the tobacco or sweet potato crops indicative of the area. Previous studies have shown that females usually value disaster preparedness more so than their male counterparts [33,42]. All of the migrant and H2A workers who took the quantitative survey resided in the same camp and were all male. Although the majority of MSFWs are male (approximately 80%), there are a significant number of females in this population [2,43]. It should be noted that the focus group participants were different demographically than the seasonal worker population which were mostly male, younger, unmarried, and did not have children. Therefore this should be noted in considering the significance of the results. Additionally, it is not possible to generalize the results of the pilot study to all Latino MSFWs. However, this was a pilot study to ensure that the instrument was culturally competent and to understand the level of awareness and barriers to utilization of community resources. The results of this study should be tested with a larger, more representative and, if possible, consistent sample. 

Another potential limitation was the social desirability of the MSFWs in the sample to answer the questions in a way they perceive as appropriate, leading to a potential response bias. As a whole, the MSFWs may have overstated their experiences or perceptions with disasters or could have exaggerated their confidence in self-reported level of preparedness because these were the answers that they felt the researchers wanted. This limitation is inherent with all self-reported data collection. To address this issue, the facilitators stressed the importance of the participants’ candid and honest responses to the questions posed. The translation of the focus groups constitutes another limitation. Although the translator was fluent in both Spanish and English and although every effort was made to provide a verbatim transcript from the audiotapes, an exact translation is difficult to ensure [44,45]. 

There were also limitations concerning the methodology of the survey: many of the workers marked more than three answers when instructed to select their top three responses and there were some questions where it appeared that the farmworkers lacked a clear understanding of the meaning. They struggled with questions that asked them to rank on a scale from one to five their level of concern that specified natural disasters could occur in the area and those involving skip patterns. In the future, finding a more effective mechanism to elicit this information would yield higher quality data. The methodology of the survey, especially since many the items involved checklists with multiple possible answers limited our ability to perform a factor analysis and other more sophisticated analytical techniques. Several of these methodological limitations were corrected in the version of the assessment instrument that was provided to the stakeholders at the conclusion of the study and that is included with this manuscript. 

The culturally competent assessment instrument developed during this study contributes to the ongoing research concerning MSFWs in the field of disaster preparedness. One of the main strengths of the instrument was that it was developed based on qualitative research within the framework of CBPR. The research team collaborated with content and cultural experts who interact with farmworkers or have significant knowledge of disaster preparedness and response to ensure that the instrument is more than a complication of existing English language surveys translated into Spanish; it was developed in large part by MSFWs specifically for their population. The methodology of the study, namely the multi-disciplinary approach using government, public, private community organization in a coordinated effort to address disaster preparedness has shown its effectiveness in the literature [37,46,47,48]. 

## 5. Conclusions

This study validates the findings of previous studies which suggest that Latinos are not prepared for a natural disaster [12,13,14,15,16,17,26]. There are several types of barriers including language difficulties, lack of knowledge, financial difficulties, logistical complications, and apathy (or one’s thinking that an event will not affect him) that impede one’s ability to be prepared for a disaster. MSFW are considered a vulnerable population and it is often those that are the most vulnerable who are disproportionally affected by disaster events [15,16,17]. Therefore, further research needs to be conducted to understand their perceptions, awareness, and unique circumstances. This study elicited the MSFWs preferences concerning preferred sources of information, preferred methods of receiving disaster preparedness information, and preferred methods of receiving alerts preceding a natural disaster event. The participants responded favorably to the idea of holding small group discussion (*platicas* or *charlas*) recommended by previous studies [22,26,37]. This information should be incorporated into disaster preparedness campaigns spearheaded by health education and outreach workers hoping to tailor their messages to this target audience [24]. 

Efforts should be made to provide disaster preparedness materials in Spanish to ensure effective preparation. Furthermore, measures should be taken to ensure that emergency alerts and news coverage concerning disaster threats be provided in Spanish. These efforts will ensure that when the next disaster strikes that emergency management professionals, outreach workers, first responders, and non-profit agencies are equipped to aid MSFWs, potentially saving lives, protecting this important agricultural workforce, and ameliorating needless suffering.

## Supplementary Files

Supplementary File 1: PDF-Document (PDF, 40 KB)
