# Supplementary material for: Assessing Disaster Preparedness among Latino Migrant and Seasonal Farmworkers in Eastern North Carolina
Source: Int J Environ Res Public Health. 2012 Aug 30;9(9):3115–33. doi: 10.3390/ijerph9093115 (PMC3499857; doi:10.3390/ijerph9093115)
Supplement: Supplementary File 1: — PDF-Document (PDF, 40 KB) [file ijerph-09-03115-s001.pdf]

OPEN ACCESS
International Journal of
Environmental Research and Public Health
ISSN 1660-4601
www.mdpi.com/journal/ijerph

Supplementary Information

Assessing Disaster Preparedness among Latino Migrant and Seasonal Farmworkers in Eastern North Carolina

Sloane Burke [1,*], Jeffrey W. Bethel [2] and Amber Foreman Britt [3]

[1] Department of Health Sciences, California State University, Northridge, CA 91330, USA
[2] College of Public Health and Human Sciences, Oregon State University, Corvallis, OR 97331, USA; E-Mail: Jeff.Bethel@oregonstate.edu
[3] BSC USAFA, United States Air Force, Colorado Springs, CO 80911, USA; E-Mail: uncamfo@gmail.com

* Author to whom correspondence should be addressed; E-Mail: sloane.burke@csun.edu; Tel.: +1-818-677-2997; Fax: +1-818-677-2045.

Received: 9 July 2012; in revised form: 6 August 2012 / Accepted: 17 August 2012 / Published: 30 August 2012

1. Qualitative Interview Guide

Focus Group Interview Questions

General

1. When you think of the words "weather-related disaster" what comes to mind?
2. What weather-related disasters are you concerned about happening here in eastern North Carolina?
3. Of these situations which one(s) do you consider yourself personally at risk for?

Prompt: Which situations are your family (if here in eastern North Carolina) personally at risk for?

4. Have you experienced a weather-related disaster in your country?

Preparation

5. Are you aware of any alert system that warns us that we are in danger of an emergency? Which one? What does it say?
6. Have you received any information on how to prepare for a weather-related disaster?
7. Do you feel that preparing is useful? If so, what would motivate you to do so?
8. What do you think Latinos can do to become better informed about preparing for a weather-related disaster?
9. What barriers do Latinos have in being prepared for a weather-related disaster?
10. Do you have a specific evacuation plan in case of a weather-related disaster? What would be the first thing you would do?

Current Disaster Supplies

11. What supplies do you have in your home that would be useful if you were affected by a weather-related disaster?
12. Are there other supplies that have been recommended that you don't have? Which ones?

Resources and Barriers to Their Utilization

13. If a weather-related disaster struck, how would you seek help?

Prompts if needed: Who would you ask? Where would you go?

14. What do you perceive as the greatest problem in the aftermath of a weather-related disaster?
15. Are you aware that there are private and government aid organizations that respond to weather-related disasters? Can you name any of them?
16. Would you seek assistance from a governmental or public agency after a weather-related disaster? Is there anything that would keep you from assessing these resources?

Trusted Sources and Information

17. Whom would you trust to talk to you about disasters? What source(s) of information do you think are the most credible? Why?
18. How or in what form would you prefer to learn more about preparing for and handling a weather-related disaster?
19. If we wanted to share information on preparing for a disaster with the Latino community, what would the information consist of? What would be the best way to communication the information?
20. Do you have any additional information to share?

2. Pilot Quantitative Instrument

A natural disaster occurs when a flood, tornado, hurricane, etc. affects the environment, and leads to financial, environmental and/or human losses. Planning beforehand and preparing one's household before a disaster strikes can help to mitigate the effects and damage in the wake of an incident.

The following questions concern your personal experience with natural disasters.

1. Have you or any of your family members experienced any of the following natural disasters?

   Check all that apply:

   ☐ Hurricane
   ☐ Extreme Heat
   ☐ Drought
   ☐ Earthquake
   ☐ Flood
   ☐ Wild Fire
   ☐ Tornado
   ☐ Winter Storm
   ☐ Other: ___________________________
   ☐ I have not experience any natural disasters.

2. Were you in North Carolina when Hurricane Floyd struck in 1999? If no skip to question 7.
   Check one:
   - ☐ Yes
   - ☐ No
   - ☐ Not sure

3. Did you lose electric power?
   Check one:
   - ☐ Yes
   - ☐ No
   - ☐ Not sure

4. Did you lose running water?
   Check one:
   - ☐ Yes
   - ☐ No
   - ☐ Not sure

5. Did you go to a shelter?
   Check one:
   - ☐ Yes
   - ☐ No
   - ☐ Not sure

6. Did your residence flood during the hurricane?
   Check one:
   - ☐ Yes
   - ☐ No
   - ☐ Not sure

7. How concerned are you about the following natural disasters affecting your community?

Type of Disaster	*Rank responses from 1 to 5. 5 = Extremely concerned, 4 = Very concerned, 3 = Concerned 2 = Somewhat concerned, 1 = Not concerned.*<br><br>*Circle the number of your choice for each.*
Hurricane	5 4 3 2 1
Extreme Heat	5 4 3 2 1
Drought	5 4 3 2 1
Earthquake	5 4 3 2 1
Flood	5 4 3 2 1
Wild Fire	5 4 3 2 1
Tornado	5 4 3 2 1
Winter Storm (snow or ice)	5 4 3 2 1

8. If you have a cell phone, do you have service at your home?
Check one:
- ☐ Yes
- ☐ No
- ☐ I don’t have a cell phone.

9. If you have a cell phone, do you have service at your work site?
Check one:
- ☐ Yes
- ☐ No
- ☐ I don’t have a cell phone.

10. Do you have a computer with access to the Internet at home?
Check one:
- ☐ Yes
- ☐ No
- ☐ I don’t have a computer with access to the Internet.

11. How well prepared do you feel your household is to handle a natural disaster?
Check one:
- ☐ Well prepared
- ☐ Somewhat prepared
- ☐ Not prepared at all
- ☐ Not sure

12. If you don’t feel prepared for a natural disaster, which of the following are the major reasons?
Check all that apply.
- ☐ Don’t have room in my home to store disaster supplies
- ☐ Don’t want to think about preparing
- ☐ Takes too much time to become prepared
- ☐ Takes too much money to become prepared
- ☐ Feel that preparing would be not be effective
- ☐ It’s not high on my list of priorities
- ☐ Don’t know how to prepare
- ☐ Don’t think an disaster will occur here
- ☐ Other ____________________________
- ☐ I feel that I am well prepared.

13. Do you have the following items?
Check all that apply.
- ☐ 3-day supply of water (1 gallon of water per person per day) for everyone who lives there
- ☐ 3-day supply of non-perishable food for everyone who lives there
- ☐ 3-day supply of medicine for each family member who requires medication
- ☐ Battery operated radio
- ☐ Batteries
- ☐ Flashlight
- ☐ First Aid Kit
- ☐ Generator
- ☐ Blankets
- ☐ I don’t have any of these items

14. If you feel that preparing for a natural disaster is important, what would motivate you?

Check all that apply.

☐ Keeping my family safe
☐ Keeping myself safe
☐ Protecting my home and belongings
☐ Other ___________________________
☐ I don't feel that it's important to prepare._

15. In the event of a natural disaster, what is your preferred source (person or organization) of emergency information? Please check your top THREE choices.

*Check your **top THREE choices**.*

☐ Police
☐ Firefighters
☐ Grower
☐ American Red Cross
☐ School
☐ Churches
☐ Latino Festivals
☐ Public Health Department
☐ Clinic/Outreach worker
☐ FEMA/government
☐ Other ___________________________

16. In the event of a natural disaster, what is your preferred *method* of receiving emergency alert information? Please check your top **THREE** choices.

*Check your **top THREE choices**.*

☐ Television
☐ Radio
☐ Internet
☐ Print Media
☐ Neighbors
☐ Friends/Family
☐ Cell phones/text alerts
☐ Other: ___________________________

17. What do you believe are potential barriers/problems to accessing emergencies services and information?

Check all that apply:

☐ Lack of information in Spanish
☐ Lack of knowledge of what to do
☐ Lack of knowledge of current location
☐ Lack of knowledge where the natural disaster is located and/or where it is heading
☐ Lack of transportation
☐ Other: ___________________________
☐ I don't believe that I will experience problems.

18. If an evacuation order was given would you evacuate?

Check one:

- ☐ Yes (If yes, skip to questions 32)
- ☐ No
- ☐ Not sure

19. If you responded No or Not Sure to question 30, why would you be unlikely to evacuate? Please select your top THREE choices.

*Check your **top THREE choices**.*

- ☐ Have special needs/medical condition that prevents evacuating
- ☐ Home could withstand the event
- ☐ Lack of transportation to a shelter
- ☐ Dislike of crowds
- ☐ Lack of trust in government or public officials
- ☐ Concern about food/water/supplies in shelter
- ☐ Don’t know location of shelter
- ☐ Concern about leaving pets
- ☐ Concern about crime/danger
- ☐ Other: ___________________

20. Have you received information about how to make your family and home safer from natural disasters?

Check one:

- ☐ Yes
- ☐ No

21. If you would like to receive disaster preparedness information, what is the best method to use? Please check your top THREE choices.

*Check your **top THREE choices**.*

- ☐ Free classes
- ☐ Videos
- ☐ Television
- ☐ Radio
- ☐ Brochures, pamphlets, posters, and other printed materials
- ☐ Family, friends, neighbors, and colleagues
- ☐ Teachers
- ☐ Skits or dramas with emergency drills
- ☐ Other: _________________________
- ☐ I’m not interesting in receiving disaster preparedness information

22. In the event of a natural disaster, what are your top concerns? Please check your top THREE choices.

*Check your **top THREE choices**.*

- ☐ Death of a family member
- ☐ Losing your house
- ☐ Losing your belongs
- ☐ Death of pet
- ☐ Losing your job (crop failure)
- ☐ Not being able to get in touch with family
- ☐ Being overwhelmed
- ☐ Other: ___________________________
- ☐ I would not be concerned

© 2012 by the authors; licensee MDPI, Basel, Switzerland. This article is an open access article distributed under the terms and conditions of the Creative Commons Attribution license (http://creativecommons.org/licenses/by/3.0/).
